# Supplementary material for: Occurrence, Potential Sources, and Risk Assessment of Volatile Organic Compounds in the Han River Basin, South Korea
Source: Int J Environ Res Public Health. 2021 Apr 2;18(7):3727. doi: 10.3390/ijerph18073727 (PMC8038302; doi:10.3390/ijerph18073727)
Supplement: Supplementary file 1 [file ijerph-18-03727-s001.pdf]

Supplementary Materials

Article

Occurrence, Potential Sources, and Risk Assessment of Volatile Organic Compounds in the Han River Basin, South Korea

Jong Kwon Im [1,*], Soon Ju Yu [1], Sujin Kim [2], Sang Hun Kim [1], Hye Ran Noh [1] and Moon Kyung Kim [3,*]

[1] National Institute of Environmental Research, Han River Environment Research Center, 42, Dumulmeori-gil 68beon-gil, Yangseo-myeon, Yangpyeong-gun, Gyeonggi-do, 12585, Korea; ysu1221@korea.kr (S.J.Y.); haemy@korea.kr (S.H.K.); anran1@korea.kr (H.R.N.)

[2] Department of Environmental Science, Center for Reservoir and Aquatic Systems Research, Baylor University, Waco, TX 76798, USA; Sujin_Kim@baylor.edu (S.K.)

[3] Institute of Health and Environment, Seoul National University, 1 Gwanak-ro, Gwanak-gu, Seoul 08826, Korea

* Correspondence: lim-jkjk@daum.net (J.K.I.); sharom21@snu.ac.kr (M.K.K.); Tel.: +82-31-770-7240 (J.K.I.); +82-2-880-2810 (M.K.K.)

Table of Contents

Table S1. Physical and chemical properties of VOCs.

Table S2. Sampling sites information.

Table S3. Sewage/wastewater treatment plants information.

Table S4. Water quality data at the sampling sites.

Table S5. The P&T-GC/MS conditions.

Table S6. SIM parameters for the analysis of VOCs.

Table S7. Accuracy of spiked test sample measurements.

Table S8. Linearity, MDL, and LOQ for spiked test samples.

Table S9. Water quality guidelines for VOCs.

Table S10. Summary statistics for VOCs analyzed in the sampling sites at Han River Basin.

Table S11. Aquatic toxicity data and PNEC values of VOCs on aquatic organisms.

Table S1. Physical and chemical properties of VOCs.

**Compound**	**CAS no.**	**Molecular formula**	**Molecular weight (g/mol)**	**Structure**	**Density at 20°C (g/cm^3)**	**Solubility in water at 25°C (mg/L)**	**Vapor pressure at 25°C (mmHg)**	**Henrys law constant (atm-m^3/mol)**	**log Kow**
cis-1,2-Dichloroethene	156-59-2	$C_2H_2Cl_2$	96.94	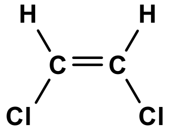	1.284	3.50×10^3	2.00×10^2	4.08×10^{-3}	1.86
trans-1,2-Dichloroethene	156-60-5	$C_2H_2Cl_2$	96.94	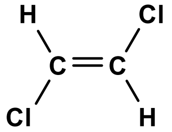	1.280	3.50×10^3	2.01×10^2	4.08×10^{-3}	1.86
cis-1,3-Dichloropropene	10061-01-5	$C_3H_4Cl_2$	110.97	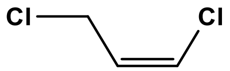	1.224	2.18×10^3	34.30	2.70×10^{-3}	2.06
trans-1,3-Dichloropropene	10061-02-6	$C_3H_4Cl_2$	110.97	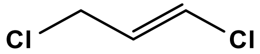	1.220	2.32×10^3	23.00	3.55×10^{-3}	2.03
Hexachlorobutadiene	87-68-3	C_4Cl_6	260.8	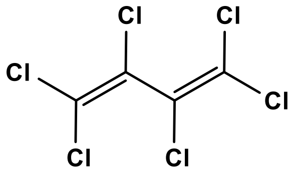	1.556	3.20	0.22	1.03×10^{-2}	4.78
Allyl chloride	107-05-1	C_3H_5Cl	76.52	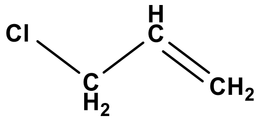	0.938	3.37×10^3	3.68×10^2	1.10×10^{-2}	1.93
Epichlorohydrin	106-89-8	C_3H_5ClO	92.52	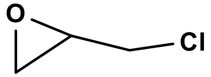	1.175	6.59×10^4	16.40	3.00×10^{-5}	0.45
1,2-Dichloropropane	78-87-5	$C_3H_6Cl_2$	112.98	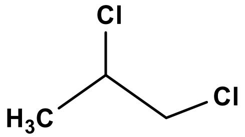	1.159	2.80×10^3	53.30	2.82×10^{-3}	1.98

Pentachloroethane	76-01-7	C_2HCl_5	202.3	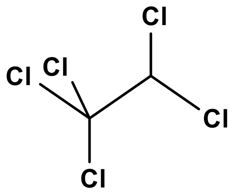	1.680	4.90×10^{2}	1.90×10^{-3}	3.50	3.22
Hexachloroethane	67-72-1	C_2Cl_6	236.7	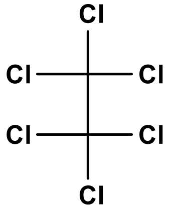	2.091	50	0.40	3.89×10^{-3}	4.14
Heptane	142-82-5	C_7H_{16}	100.2	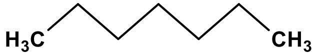	0.680	3.40	46.00	1.80	4.66
2-Methylhexane	591-76-4	C_7H_{16}	100.2	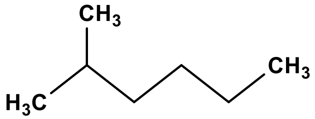	0.680	None	65.99	-	-
1-Octene	111-66-0	C_8H_{16}	112.21	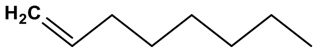	0.715	4.10	17.40	-	4.57
Nonane	111-84-2	C_9H_{20}	128.25	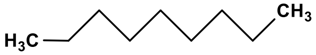	0.718	0.22	4.45	3.40	5.65
1,2-Diethylbenzene	135-01-3	$C_{10}H_{14}$	134.22	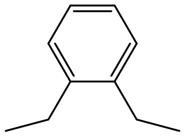	0.880	71.1	1.05	-	3.72
1,3-Diethylbenzene	141-93-5	$C_{10}H_{14}$	134.22	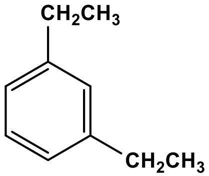	0.860	24.0	1.20	-	4.44
1,4-Diethylbenzene	105-05-5	$C_{10}H_{14}$	134.22	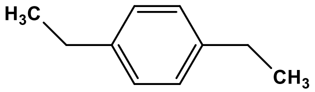	0.862	24.8	1.06	-	4.45

Kow: Octanol-water partition coefficient; Source: PubChem (https://pubchem.ncbi.nlm.nih.gov/).

Table S2. Sampling sites information.

Medium-sized watershed	Sampling sites	Location		Note
		Longitude	Latitude	
Bukhan River	BR-1	128˚11'13.69"	38˚04'58.72"	Rural
	BR-2	127˚42'49.94"	37˚52'12.22"	Rural
	BR-3	127˚31'17.13"	37˚49'33.15"	Rural
	BR-4	127˚35'34.94"	37˚43'12.09"	Rural
	BR-5	127˚25'08.08"	37˚43'41"	Rural
Namhan River	NR-1	128˚27'35.38"	37˚09'56.03"	Rural
	NR-2	127˚53'59.44"	36˚26'29.43"	Rural
	NR-3	127˚55'06.98"	37˚24'35.58"	Rural
	NR-4	127˚45'07.95"	37˚14'11.08"	Rural
	NR-5	127˚32'21.92"	37˚19'54.24"	Rural
	NR-6	127˚31'55.39"	37˚27'37.69"	Rural
	NR-7	127˚18'46.51"	37˚25'19.79"	Rural
Hantan-Imjin River	HIR-1	127˚03'28.98"	38˚00'21.86"	Rural
	HIR-2	127˚04'50.89"	38˚00'09.46"	Rural
	HIR-3	127˚02'06.78"	38˚03'12.79"	Rural
	HIR-4	126˚55'12.39"	37˚59'01.39"	Rural
Han River mainstream	HR-1	127˚10'04.98"	37˚34‘58.67"	Urban
	HR-2	127˚04'14.24"	37˚30'36.75"	Urban
	HR-3	127˚03'19.63"	37˚32'57.56"	Urban
	HR-4	127˚02'51.73"	37˚32'51.76"	Urban
	HR-5	126˚52'51.39"	37˚32'52.21"	Urban
	HR-6	126˚50'06.88"	37˚35'35.59"	Urban
	HR-7	126˚46'37.24"	37˚34'10.39"	Urban
	HR-8	126˚44'18.74"	37˚45'05.37"	Urban
Anseong Stream	AS-4	126˚58'51.08"	36˚56'56.01"	Rural
Industrial complex	ICS-1	127˚14'38.56"	37˚57'55.80"	Industrial
	ICS-2	127˚12'50.42"	38˚00'36.57"	Industrial
	ICS-3	127˚14'49.53"	38˚00'46.21"	Industrial
	ICS-4	126˚59'51.92"	37˚52'16.13"	Industrial
	ICS-5	127˚01'19.19"	37˚52'09.34"	Industrial
	ICS-6	127˚03'40.27"	37˚57'27.87"	Industrial
	ICS-7	127˚03'31.84"	37˚56'48.70"	Industrial
	ICS-8	126˚44'40.88"	37˚49'17.09"	Industrial
	ICS-9	127˚14'23.35"	37˚00'21.68"	Industrial
	ICS-10	127˚11'23.02"	36˚58'31.44"	Industrial
	ICS-11	127˚04'15.07"	37˚01'48.60"	Industrial

Table S3. Sewage/wastewater treatment plants information.

STP/WWTP			Capacity (m^3/day)	Discharge (m^3/day)	Medium-sized watershed
STP	1	Wonju STP	156,000	127,800	Namhan River
	2	Munmak STP	7,000	5,153	
	3	Gwangju STP	25,000	24,177	
	4	Konjiam STP	23,000	21,415	
	5	Yangbeol STP	20,000	10,871	
	6	Samri STP	5,000	4,549	
	7	Icheon STP	43,000	40,828	
	8	Danwol STP (Icheon)	4,000	1,608	
	9	Yongmun STP	4,700	2,968	
	10	Danwol STP (Yangpyeong)	1,700	1,461	
	11	Inje Bukmyeon STP	2,000	1,474	Bukhan River
	12	Cheongsan STP	2,100	1,198	Hantan-Imjin River
	13	Dongducheon STP	86,000	68,255	
	14	Guri STP	160,000	139,468	Han River mainstream
	15	Jingeon STP	100,000	93,431	
	16	Tancheon STP	900,000	758,962	
	17	Gwacheon STP	30,000	17,652	
	18	Seongnam STP	507,000	365,638	
	19	Jungnang STP	1,590,000	1,336,827	
	20	Anyang Bakdal STP	250,000	162,013	
	21	Seoksu STP	300,000	190,983	
	22	Gulpo STP	900,000	700,772	
	23	Geumchon STP	27,000	24,522	
	24	Samsong STP	16,000	7,910	
Industrial WWTP	25	Pyeongtaek Songtan WWTP	11,000	7,283	Anseong Stream
	26	Anseong 2nd WWTP	6,200	3,845	
	27	Paju LCD WWTP	185,000	144,304	Hantan-Imjin River
	28	Yangju Hongjuk WWTP	1,000	456	
	29	Yangju Geomjun WWTP	23,000	10,412	
	30	Pocheon Yangmun WWTP	14,000	9,728	
	31	Pocheon Jangja WWTP	18,750	4,999	

Source: South Korea Ministry of Environment, 2017 Sewerage statistics and 2017 Operation status of wastewater treatment plants.

Table S4. Water quality data at the sampling sites.

Sampling sites	Water temp. (°C)	pH	DO (mg/L)	Cond. (μS/cm)
BR-1	16.87 ± 6.82	7.66 ± 0.71	9.76 ± 0.90	94.00 ± 34.51
BR-2	20.27 ± 5.83	7.92 ± 0.73	10.16 ± 1.23	187.33 ±15.37
BR-3	18.50 ± 6.32	7.75 ± 0.20	10.26 ± 0.88	127.33 ±59.72
BR-4	21.73 ± 6.90	8.18 ± 0.32	9.55 ± 0.89	173.67 ± 86.67
BR-5	19.67 ± 6.97	8.07 ± 0.30	10.41 ± 1.06	178.00 ± 62.38
NR-1	22.30 ± 7.59	8.35 ± 0.56	10.47 ± 1.50	234.00 ± 55.11
NR-2	25.10 ± 3.64	7.39 ± 1.09	9.95 ± 3.07	232.67 ± 66.46
NR-3	21.90 ± 2.78	7.22 ± 0.60	6.97 ± 1.63	462.00 ± 123.72
NR-4	23.07 ± 5.72	8.38 ± 0.59	11.35 ± 2.62	281.67 ± 137.61
NR-5	23.27 ± 4.77	7.70 ± 0.20	8.03 ± 0.68	833.33 ± 451.19
NR-6	21.50 ± 6.07	8.28 ± 0.43	10.62 ± 1.31	178.33 ± 76.51
NR-7	21.63 ± 4.54	7.77 ± 0.25	8.98 ± 1.61	341.33 ± 193.98
IH-1	23.00 ± 2.10	7.31 ± 0.18	6.60 ± 0.90	494.33 ± 138.02
IH-2	22.73 ± 2.45	6.92 ± 0.23	5.67 ± 2.37	447.33 ± 183.92
IH-3	21.13 ± 3.60	7.07 ± 0.79	7.75 ± 2.22	196.67 ± 45.54
IH-4	22.83 ± 2.31	6.91 ± 0.32	6.26 ± 1.04	402.33 ± 204.78
IH-5	22.57 ± 2.83	6.99 ± 0.71	6.64 ± 0.78	532.33 ± 234.63
IH-6	21.23 ± 3.62	6.97 ± 0.61	6.62 ± 1.91	396.67 ± 91.57
IH-7	21.63 ± 2.98	7.11 ± 0.45	5.79 ± 2.60	210.67 ± 23.03
IH-8	22.83 ± 4.38	7.34 ± 0.42	8.83 ± 2.38	595.33 ± 297.52
HIR-1	24.17 ± 4.97	8.10 ± 0.41	9.99 ± 1.34	2051.00 ± 1464.72
HIR-2	23.53 ± 5.18	8.82 ± 1.07	12.73 ± 3.52	485.33 ± 387.60
HIR-3	23.37 ± 4.55	8.39 ± 1.56	13.48 ± 4.55	456.00 ± 329.55
HIR-4	24.30 ± 5.86	7.95 ± 1.30	12.03 ± 3.25	166.33 ± 10.26
AS-1	23.70 ± 4.10	7.53 ± 0.74	8.34 ± 1.66	895.67 ± 500.50
ICS-1	24.40 ± 7.48	8.10 ± 0.40	10.71 ± 1.84	996.33 ± 885.85
ICS-2	25.93 ± 6.39	8.12 ± 0.46	11.00 ± 1.78	1083.33 ± 834.80
ICS-3	22.50 ± 6.11	8.64 ± 0.68	11.22 ± 1.77	228.00 ± 77.95
ICS-4	21.77 ± 6.43	7.82 ± 0.46	7.50 ± 0.76	2027.33 ± 1392.42
ICS-5	22.57 ± 6.37	7.72 ± 0.15	5.98 ± 1.28	1941.67 ± 1422.76
ICS-6	24.67 ± 6.19	7.86 ± 0.40	9.88 ± 2.74	2133.67 ± 1267.58
ICS-7	24.87 ± 4.11	7.63 ± 0.09	8.54 ± 0.51	2578.00 ± 1275.29
ICS-8	28.40 ± 1.25	7.98 ± 0.81	7.93 ± 0.06	1323.00 ± 69.94
ICS-9	22.50 ± 1.56	7.48 ± 0.08	7.64 ± 1.52	526.00 ± 417.61
ICS-10	25.33 ± 7.32	7.14 ± 0.83	9.60 ± 2.26	804.67 ± 745.92
ICS-11	22.73 ± 1.46	7.13 ± 0.74	5.90 ± 2.00	517.33 ± 287.51

Table S5. The P&T-GC/MS conditions.

**P&T conditions**		
	Valve oven temperature	150 ℃
	Transfer line temperature	150 ℃
	Syringe fill volume	25 mL
	Sample mount temperature	90 ℃
	Purge	He, 40 mL/min, 0℃ for 11 min
	Desorb	200 mL/min, 250 ℃ for 2 min (preheat temp. 245 ℃)
	Bake	400 mL/min, 260 ℃ for 10 min
**GC/MS conditions**		
GC	Column	Agilent DB-624 column (60 m × 0.32 mm × 1.8 μm)
	Carrier gas flow	He at 1.0 mL/min constant flow
	Injector	temperature 250ºC, Splitless mode
	Oven temperature	30 ºC for 2 min
		8 ºC/min to 140 ºC, hold 10 min
		30 ºC/min to 220 ºC, hold 4 min
MS	Ionization mode	Electron Ionization (EI)
	Electron Energy	70 eV
	Source temperature	200 ºC
	Transfer line temperature	250 ºC
	Data Acquisition	Selected Ions Monitoring (SIM)

Table S6. SIM parameters for the analysis of VOCs.

Compound		t_R (min)	QI (m/z)	CI (m/z)	
Target analytes	cis-1,2-Dichloroethene	12.052	60.9	95.8	62.9
	trans-1,2-Dichloroethene	9.945	60.9	95.8	62.9
	cis-1,3-Dichloropropene	17.066	74.9	39.1	76.8
	trans-1,3-Dichloropropene	18.265	74.9		
	Hexachlorobutadiene	30.523	224.7	190.0	260.0
	Allyl chloride	9.118	41.1	39.1	
	Epichlorohydrin	16.981	57.0	49.0	27.2
	1,2-Dichloropropane	15.596	62.9	76.0	61.9
	Pentachloroethane	25.291	116.7	118.7	166.7
	Hexachloroethane	27.219	118.7	116.7	201.0
	Heptane	14.082	43.1	71.0	57.0
	2-Methylhexane	12.845	43.1	57.0	85.0
	1-Octene	17.609	70.0	55.0	43.1
	Nonane	21.181	43.1	57.0	85.0
	1,2-Diethylbenzene	26.821	104.9	118.9	134.0
	1,3-Diethylbenzene	26.400	118.9	104.8	134.0
	1,4-Diethylbenzene	26.570	104.9	119.0	134.0
Internal standards	Fluorobenzene	14.320	95.8		
	1,4-Dichlorobenzene-d4	25.993	149.8		
	Chlorobenzene-d5	20.860	116.9		

t_R: Retention time; MM: Monoisotopic Mass; QI: Quantification ion; CI: Confirmation ion.

Table S7. Accuracy of spiked test sample measurements.

Compound	Spiked (μg/L)	Measured (μg/L)	R %	RSD %
cis-1,2-Dichloroethene	0.1500	0.1290 ± 0.0018	86.0	1.4
trans-1,2-Dichloroethene	0.1500	0.1741 ± 0.0030	98.1	2.0
cis-1,3-Dichloropropene	0.1500	0.1270 ± 0.0048	84.7	3.8
trans-1,3-Dichloropropene	0.1500	0.1155 ± 0.0029	77.0	2.5
Hexachlorobutadiene	0.1000	0.0754 ± 0.0022	75.4	2.9
Allyl chloride	0.1000	0.0967 ± 0.0089	96.7	9.2
Epichlorohydrin	3.0000	2.7729 ± 0.1225	92.4	4.4
1,2-Dichloropropane	0.1500	0.1416 ± 0.0023	94.4	1.6
Pentachloroethane	0.5000	0.5069 ± 0.0792	101.4	15.6
Hexachloroethane	0.1000	0.0882 ± 0.0041	88.2	4.6
Heptane	0.3000	0.2708 ± 0.0219	90.3	8.1
2-Methylhexane	0.3500	0.3834 ± 0.0407	109.5	10.6
1-Octene	0.3000	0.2811 ± 0.0180	93.7	6.4
Nonane	0.2000	0.2108 ± 0.0125	105.4	5.9
1,2-Diethylbenzene	0.1000	0.0897 ± 0.0031	89.7	3.4
1,3-Diethylbenzene	0.1000	0.0925 ± 0.0033	92.5	3.6
1,4-Diethylbenzene	0.1000	0.0889 ± 0.0025	88.9	2.9

R: Average recovery; RSD: Relative standard deviation; R^2: Coefficient of determination.

Table S8. Linearity, MDL, and LOQ for spiked test samples.

Compound	Slope	Intercept	R^2	Linearity range (μg/L)*	MDL (μg/L)	LOQ (μg/L)
cis-1,2-Dichloroethene	5.8138	-0.0225	0.9959	0, 0.0025 ~ 0.3000	0.0020	0.0064
trans-1,2-Dichloroethene	5.4445	0.0015	0.9993	0, 0.0025 ~ 0.2000	0.0015	0.0048
cis-1,3-Dichloropropene	3.1828	-0.0016	0.9983	0, 0.0050 ~ 0.2000	0.0036	0.0115
trans-1,3-Dichloropropene	2.5297	0.0155	0.9993	0, 0.0100 ~ 0.5000	0.0030	0.0097
Hexachlorobutadiene	6.0556	-0.0125	0.9994	0, 0.0050 ~ 0.3000	0.0004	0.0012
Allyl chloride	3.1070	-0.0177	0.9970	0, 0.0100 ~ 0.3000	0.0085	0.0271
Epichlorohydrin	0.0277	-0.0008	0.9938	0, 0.2000 ~ 5.0000	0.0944	0.3007
1,2-Dichloropropane	5.5023	-0.0022	0.9987	0, 0.0025 ~ 0.3000	0.0011	0.0034
Pentachloroethane	0.4902	0.0020	0.9997	0, 0.0100 ~ 1.0000	0.0023	0.0072
Hexachloroethane	13.5897	-0.0564	0.9991	0, 0.0050 ~ 0.3000	0.0024	0.0076
Heptane	1.1031	-0.0170	0.9962	0, 0.0200 ~ 1.0000	0.0074	0.0237
2-Methylhexane	1.4075	-0.0087	0.9985	0, 0.0200 ~ 0.5000	0.0016	0.0052
1-Octene	0.6936	0.0042	0.9983	0, 0.0200 ~ 0.5000	0.0055	0.0175
Nonane	0.3940	0.0039	0.9994	0, 0.0200 ~ 1.0000	0.0029	0.0093
1,2-Diethylbenzene	9.4352	0.0151	0.9998	0, 0.0025 ~ 0.3000	0.0017	0.0053
1,3-Diethylbenzene	8.2793	0.0317	0.9997	0, 0.0025 ~ 0.5000	0.0017	0.0053
1,4-Diethylbenzene	8.3739	0.0287	0.9998	0, 0.0100 ~ 0.3000	0.0015	0.0046

* Seven calibration standards were pretreated and analyzed.

R^2: Coefficient of determination; MDL: Method detection level; LOQ: Limit of quantitation.

Calculation of MDL and LOQ:

- MDL = SD × t-value
- LOQ = SD × 10

where, SD: standard deviation of seven replicate measurements; t-value: student's t value with six degrees of freedom (3.143).

Table S9. Water quality guidelines for VOCs.

Compound	Ambient water (μg/L)			Drinking water (μg/L)			
	South Korea	US EPA	Canada	US EPA		Japan	WHO
				MCLG	MCL		
cis-1,2-Dichloroethene	30	5	-	70	70	40	50
trans-1,2-Dichloroethene	30	5	-	100	100	40	50
cis-1,3-Dichloropropene	-	0.2	-	-	-	-	-
trans-1,3-Dichloropropene	-	0.2	-	-	-	-	-
Hexachlorobutadiene	-	0.046	1.3	0.11	0.11	-	0.5
Allyl chloride	-	-	-	-		-	-
Epichlorohydrin	-	-	-	zero	TT[1]	-	-
1,2-Dichloropropane	-	0.52	-	zero	5	-	-
Pentachloroethane	-	440	-	-	-	-	-
Hexachloroethane	-	62	-	-	-	-	-
Heptane	-	-	-	-	-	-	-
2-Methylhexane	-	-	-	-	-	-	-
1-Octene	-	-	-	-	-	-	-
Nonane	-	-	-	-	-	-	-
Diethylbenzenes-total	-	-	-	-	-	-	-

MCLG: Maximum contaminant level goal; MCL: Maximum contaminant level; TT; Treatment Technique. A required process intended to reduce the level of a contaminant in drinking water.

[1] When epichlorohydrin is used in drinking water systems, the combination (or product) of dose and monomer level shall not exceed that equivalent to an epichlorohydrin-based polymer containing 0.01% monomer dosed at 20 mg/L.

Table S10. Summary statistics for VOCs analyzed in the sampling sites at the Han River Basin

Sampling Sites	Mean Conc. (μg L^{-1})	Min Conc. (μg L^{-1})	Max Conc. (μg L^{-1})	Total Conc. (μg L^{-1})	Detection Frequency (%)
BR-1	0.0040	0.0006	0.0073	0.0079	3.92
BR-2	0.0096	0.0011	0.0180	0.0191	3.92
BR-3	0.0047	0.0047	0.0047	0.0047	1.96
BR-4	0.0015	0.0015	0.0015	0.0015	1.96
BR-5	0.0028	0.0028	0.0028	0.0028	1.96
NR-1	0.0065	0.0014	0.0116	0.0130	3.92
NR-2	NA	NA	NA	NA	NA
NR-3	0.0070	0.0037	0.0103	0.0141	3.92
NR-4	0.0066	0.0017	0.0114	0.0131	3.92
NR-5	0.0354	0.0074	0.0971	0.1418	7.84
NR-6	0.0026	0.0026	0.0026	0.0026	1.96
NR-7	0.0088	0.0022	0.0197	0.0438	9.80
HR-1	0.0045	0.0015	0.0117	0.0272	11.76
HR-2	0.0061	0.0016	0.0105	0.0182	5.88
HR-3	0.0051	0.0029	0.0081	0.0254	9.80
HR-4	0.0149	0.0019	0.0458	0.0746	9.80
HR-5	0.0875	0.0037	0.2205	0.6122	13.73
HR-6	0.0066	0.0021	0.0159	0.0332	9.80
HR-7	0.0241	0.0046	0.0509	0.1689	13.73
HR-8	0.0091	0.0041	0.0152	0.0455	9.80
HIR-1	0.4308	0.0023	1.8131	3.0153	13.73
HIR-2	0.0961	0.0057	0.3240	0.3842	7.84
HIR-3	0.0246	0.0026	0.0989	0.1473	11.76
HIR-4	0.0103	0.0014	0.0279	0.0309	5.88
AS-1	0.0250	0.0056	0.0903	0.1497	11.76
ICS-1	0.0215	0.0069	0.0487	0.1288	11.76
ICS-2	0.1095	0.0052	0.2873	0.5473	9.80
ICS-3	0.0124	0.0023	0.0281	0.0494	7.84
ICS-4	0.0542	0.0027	0.1367	0.3791	13.73
ICS-5	0.0388	0.0018	0.0878	0.3101	15.69
ICS-6	0.0896	0.0035	0.1898	0.5378	11.76
ICS-7	0.0696	0.0068	0.1634	0.4174	11.76
ICS-8	0.0154	0.0017	0.0268	0.0616	7.84
ICS-9	0.0490	0.0016	0.1056	0.3922	15.69
ICS-10	0.0176	0.0027	0.0348	0.0881	9.80
ICS-11	0.0564	0.0164	0.1700	0.3383	11.76

NA: Not Applicable.

Table S11. Aquatic toxicity data and PNEC values of VOCs on aquatic organisms.

**Compound**	**Species**	**Effect**	**Parameter**	**Conc (mg/L)**	**Test Duration (Days)**	**Reference**	**AF[a]**	**PNEC (μg/L)**
cis-1,2-Dichloroethene	*Pseudokirchneriella subcapitata*	Population	EC_{50}	59.69	2	Tsai and Chen (2007)	1000	59.7
trans-1,2-Dichloroethene	*Pseudokirchneriella subcapitata*	Population	EC_{50}	36.36	2	Tsai and Chen (2007)	1000	36.4
Hexachlorobutadiene	*Pimephales promelas*	Mortality	NOEC	0.0065	NR	Geiger et al. (1985)	50	0.130
1,2-Dichloropropane	*Pimephales promelas*	Growth	NOEC	6	32	Benoit et al. (1982)	50	120

Aquatic toxicity data was collected from the US EPA ECOTOX database (http://cfpub.epa.gov/ecotox). Due to the limited ecotoxicological information, PNEC derivation and subsequent RQ calculation for 1,3-diethylbenzene, 1,4-diethylbenzene, and heptane were not performed.

[a]Assessment Factor was determined according to the technical guidance of European Commission (2018).

References

Benoit D, Puglisi F, Olson D. 1982. A fathead minnow pimephales promelas early life stage toxicity test method evaluation and exposure to four organic chemicals. Environmental Pollution Series A, Ecological and Biological 28:189-197.

European Commission (EC), 2018. Technical Guidance Document for Deriving Environmental Quality Standards (TGD-EQS).

Geiger DL, Northcott CE, Call DJ, Brooke LT. 1985. Acute Toxicities of Organic Chemicals to Fathead Minnows (*Pimephales promelas*), Volume II. Center for Lake Superior Environmental Studies, University of Wisconsin, Superior, WI:326 p.

Tsai KP, Chen CY. 2007. An algal toxicity database of organic toxicants derived by a closed-system technique. Environmental Toxicology and Chemistry: An International Journal 26:1931-1939.
